# Surface Roughening of Electrolyte Membrane for Pt- and Ru-Sputtered Passive Direct Methanol Fuel Cells

**DOI:** 10.3390/ma12233969

**Published:** 2019-11-29

**Authors:** Wonyeop Jeong, Gu Young Cho, Suk Won Cha, Taehyun Park

**Affiliations:** 1 School of Mechanical and Aerospace Engineering, Seoul National University, 1 Gwanak-ro, Gwanak-gu, Seoul 08826, Korea; starryshot@snu.ac.kr; 2 Department of Mechanical Engineering, Dankook University, 152, Jukjeon-ro, Suji-gu, Yongin-si, Gyeonggi-do 16890, Korea; guyoungcho@dankook.ac.kr; 3 School of Mechanical Engineering, Soongsil University, 369 Sangdo-ro, Dongjak-gu, Seoul 06978, Korea

**Keywords:** direct methanol fuel cells, sputter, sandpaper, roughness, electrochemical impedance spectroscopy, polarization

## Abstract

Platinum (Pt) and ruthenium (Ru) were sputtered on an electrolyte membrane and it was used as a membrane-electrode assembly for passive direct methanol fuel cells (DMFCs) operating with high concentration methanol solution (4 M). Thick (Pt of 300 nm and Ru of 150 nm) and thin (Pt of 150 nm and Ru of 75 nm) sputtered catalysts were prepared and their performance was first evaluated to find out the best sputtering conditions showing higher performance. Subsequently, four electrolyte membranes with different surface roughness were prepared to investigate its influence on the performance. As a result, the performance of the passive DMFC showed increasing tendency as the roughness is low, while the performance was decreased as the roughness was high, indicating there exists an optimal roughness of the electrolyte membrane. It was further investigated through morphological study through electron microscopy that such performance variation is attributed to the surface of sputtered Pt–Ru catalyst on the rough electrolyte membrane that adequate roughness induces the increase of reactive area while a too rough surface bears the poor contact of it with gas-diffusion layer.

## 1. Introduction

Many researchers have been working on the investigation of the alternatives of current market-leading lithium-ion batteries to store more energy in a limited volume. One of the technologies is considered as a fuel cell because fuel cells have potentially higher energy density than lithium-ion batteries [1,2,3]. In addition, fuel cells have other advantageous characteristics such as scalability, eco-friendliness, high efficiency, and no need to secure recharging time [4,5,6,7]. That is why many portable fuel cell prototypes are actively coming out to markets for the purpose of replacing batteries [8,9,10].

Among various fuel cell types, polymer electrolyte membrane fuel cells (PEMFCs) are the most famous type because they operate at low temperature (<100 °C) and their technological level is close to commercialization level. Fuel cell electric vehicles, drones including unmanned aerial vehicles and quadrotors, and power plants already manufactured or installed prove it [11,12,13,14]. Here, it is noticeable that the recent world-champion record of the flight time of quad-/hexa-rotors exceeds 12 h and it has been enabled by employing PEMFCs with liquid hydrogen storage [15]. Comparing it with the fact that the top-flight time by any batteries is shorter than 30 min, the fuel cells and their features they could enable are amazing.

The use of hydrogen as a fuel, however, bears a problem: The hydrogen storage technology is not sufficiently matured than fuel cells [16,17,18]. In addition, hydrogen storage is directly related to safety, so it makes the PEMFCs still distant from complete commercialization. In order to overcome this, direct methanol fuel cells (DMFCs) have been investigated for decades. The basic theory and the structure of operation are the same as PEMFCs, but they are distinguished in that a methanol solution instead of hydrogen is supplied and ruthenium (Ru) as well as platinum (Pt) is normally added in an electro-catalyst. Here, if the chemical energy resource is stored as a methanol, it is enabled theoretically that the DMFC systems can store more energy than normal PEMFC systems in a same volume.

The problems of the DMFCs are that they require more novel metals (Pt and Ru) than PEMFCs, the resulting electrochemical performance is still lower than PEMFCs, and their system is highly complicated. The third problem comes from the feature that the water management and resulting methanol concentration within the system is highly important to maximize the performance and secure the long-term durability. One way to resolve this problem is to simplify the fuel supply system in an anode and cathode by designing them “fully passive”. That is, the supply of whole reactants in DMFC fully depends on free convection. Many reports about this fully passive DMFCs are already in literature [2,19,20,21,22,23,24,25,26,27].

In this study, we especially employed the sputtering method to fabricate the membrane-electrode assembly (MEA) for passive DMFCs. It is because two advantages could be achieved from this approach: Lowering the use of novel metals and drive down the manufacturing cost of DMFCs. It is because the sputtering method has been experimentally proved to reduce the use of novel metals. Although most of sputtering-based fuel cells are about PEMFCs [28,29,30,31,32], we expected that the application of this technique to DMFCs would result in the same effect as PEMFCs. In addition, industrial infrastructure of sputtering is already tremendous due to the development of semiconductors industry. It means that we could maximize the advantages of the development of this sputter-based MEA fabrication approach for passive DMFCs. Unfortunately, to our knowledge, no report about this sputtered catalyst-based passive DMFCs can be found in literature. With this sputtering approach, this study also investigates the effect of roughness of the electrolyte membrane on the performance of the passive DMFCs. The MEAs with four roughnesses were prepared and their micro-morphologies are investigated through scanning-electron microscopy (SEM). Moreover, the thickness of the sputtered Pt and Ru is tested to find out the structural effect of sputtered layers (Note too thin catalyst would not activate the oxidation of the methanol while too thick could disturb the diffusion of reactants.). Finally, the performances of the passive DMFCs with four MEAs are measured and compared to find out the optimal roughness showing the best electrochemical performance and the relation between the roughness of an electrolyte membrane and the resulting performance.

## 2. Materials and Methods

**Figure 1:**
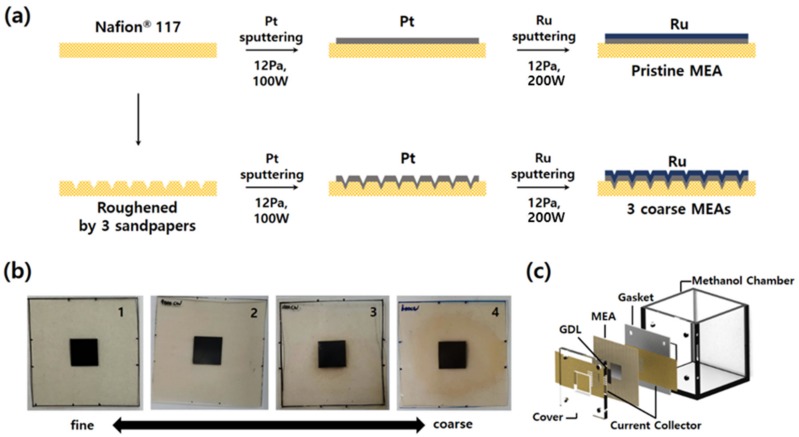
(**a**) Schematic of the fabrications of the membrane-electrode assembly (MEAs) with different roughnesses. (**b**) Real images of as-prepared MEAs. (**c**) Exploded image of the fully passive direct methanol fuel cells (DMFC) used in this study. This is a figure, Schemes follow the same formatting.

Four types of MEAs were fabricated to compare the effects of surface roughness of an electrolyte membrane on electrochemical characteristics of passive DMFCs, as shown in Figure 1a. First, the standard MEA with a pristine electrolyte membrane (Nafion^®^ 117, DuPont Co., Midland, MI, USA) was fabricated using sputter. Pt and Ru were deposited sequentially on the anode side of Nafion^®^ 117 to deposit bi-layered catalyst. 100 and 200 W of DC sputtering power were applied to Pt and Ru target, respectively. The sputtering conditions were 12.0 Pa of Ar gas pressure and room temperature of substrate in all cases. Two bi-layered catalysts were fabricated first: Thicknesses of Pt–Ru catalysts of 300 and 150 nm, and 150 and 75 nm, respectively. These two samples were characterized and the thickness showing the best performance was selected. After depositing bi-layered Pt–Ru catalysts, cathodic catalyst layers were deposited on the other side of the MEA by sputtering again. Pt was deposited with 100 W of DC power and 12.0 Pa of Ar pressure. The electrochemically reactive area was precisely controlled and defined by using the physical masking tape with a hole of 1 × 1 cm^2^. In the fabrication of the MEAs with rough electrolyte surfaces, Nafion^®^ 117 membrane was rubbed with sandpapers to change the surface roughness. Three kinds of sandpapers (600, 2000, and 4000 grit, Daesung Abrasive Co., Yonki, Chungnam, Korea) with different roughness were used to vary the surface roughness. When the membrane is roughened, its surface becomes opaque. We scrubbed the center of the membrane until it became uniformly opaque over a wider range than the catalysts deposition area. After preparing the membrane with three different roughnesses, Pt–Ru catalysts were deposited using the same sputtering conditions as described above. Digital camera images of the as-prepared four MEAs are indicated in Figure 1b.

After preparing MEAs, the four-step pretreatment process was performed to secure the high protonic conductivity of the electrolyte membrane. This process is intended to remove impurities and recover the sulfonic acid group in the Nafion^®^ 117 membrane. The procedure was as follows: (1) Boiling for 1 h in 5 vol% H_2_O_2_ solution, (2) boiling for 1 h in deionized water, (3) boiling for 1 h in 0.5 M H_2_SO_4_ solution, and (4) boiling for 1 h in deionized water [33].

Electrochemical characterizations of the as-fabricated MEAs and the passive DMFCs comprising thereof were conducted using the custom-made passive DMFC setup, as shown in Figure 1c. It consists of a methanol chamber, two polytetrafluoroethylene gaskets, two Au-coated stainless-steel current collectors, two gas-diffusion layers (GDLs, Sigracet 39BC, SGL Carbon Co., Wiesbaden, Germany) with micro-porous layers at one side, and one endplate. All components were assembled tightly by four bolts and nuts. The 0.1 mm thick current collectors were fabricated by laser-cutting and Au was subsequently sputtered on one side of it. For Au deposition, 200 W of DC power and 0.67 Pa of Ar pressure at room temperature were used. The thickness of the as-deposited Au layer was 20 nm. The volume of methanol chamber was 5 × 5 × 5 cm^3^ and a 4 M methanol solution was supplied as a fuel into the chamber.

The surface and cross-sectional images of the as-prepared MEAs were obtained and investigated using a field-emission scanning-electron microscope (FE-SEM, Zeiss Supra 55VP, Carl Zeiss, Oberkochen, Germany) and a focused-ion beam SEM (FIB-SEM, Nova 600, FEI Company, Hilsboro, OR, USA). In the FIB process, a platinum buffer layer was deposited to protect the electrode layer before milling. The main trench was milled with an ion current of 1 nA and a lower current of 0.5 nA was used to polish the cross-section for imaging. The performance measurement was carried out at room temperature using a potentiostat (Reference 600, Gamry Instruments Inc., Warminster, PA, USA). Electrodes of MEAs were simply exposed to the 4 M methanol and ambient air, respectively. Thus, fuel and air were supplied to each electrode by free convection, not forced nor controlled. Polarization characteristics were investigated by measuring current–voltage (I–V) curves. The measurement started from the open-circuit voltage (OCV) of the fuel cell, and swept towards 0.1 V. The voltage sweep speed was 0.01 V/s, and resulting current was monitored. It was halted at 0.1 V compared to RHE (reversible hydrogen electrode). Electrochemical impedance spectra (EIS) were measured and visualized on Nyquist plot. A sinusoidal voltage input with an amplitude of 0.01 V and a frequency range from 10^6^ to 0.1 Hz at 0.1 V compared to RHE was applied to the fuel cell and the resulting current response was measured.

## 3. Results and Discussion

**Figure 2:**
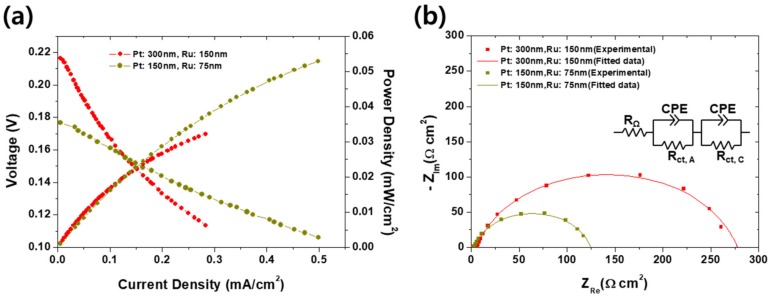
(**a**) Current–voltage and current–power curves, and (**b**) electrochemical impedance spectra (EIS) measured at 0.1 V compared to reversible hydrogen electrode (RHE) corresponding to (**a**) of the passive DMFCs with anodic catalysts of 150 nm thick Ru on 300 nm thick Pt and 75 nm thick Ru on 150 nm thick Pt.

The effect of the thickness of an anodic catalyst layer on the electrochemical performance of the passive was first investigated, as shown in Figure 2a, b. It is because too thin catalyst cannot activate the polarization while too thick could disturb the diffusion of the methanol and air. Same phenomena can be found elsewhere that the PEMFCs should be clamped with appropriate pressure because too strong clamping pressure could stuff the pores inside GDLs, thereby resulting in more frequent flooding and high activation overvoltage by the lowered concentration of the reactants near reactive sites. On the other hand, weak clamping force could bear the high contact resistance between GDLs and bipolar plates [34]. It is speculated that this can be compared by the experimental results in Figure 2 because the phenomena and resulting performance variation resemble: Too thick catalyst could disturb the diffusion of reactants. In real, as shown in Figure 2a, the DMFC with a thin catalyst (75 nm thick Ru on 150 nm thick Pt) shows higher peak power density (0.53 mW/cm^2^) than that with thick (0.33 mW/cm^2^) one. Although both power curves are apparently in increasing tendency so we could not mention a “peak” power density, it seems the DMFC with thicker catalyst could not outperform the thin one at high current density region. It is thought that such difference is attributed to the disturbance of mass transport by the thick catalyst. According to the EIS results indicated in Figure 2b, it is no doubt that charge transport resistance is a dominant factor of the final electrochemical performance because in both cases, the charge transport resistance (122–275 Ω·cm^2^) is extremely higher than the ohmic resistance (<5 Ω·cm^2^). In addition, the charge transfer resistance of the DMFC with thick catalyst is higher than that with thin catalyst. If considering the activation overvoltage is described by the Butler–Volmer behavior, increasing charge transfer resistance with the increasing Pt cannot be explained. In this case, it is speculated that the stuffed pores by thicker layer inactivated the catalyst. It corresponds with the observation in Figure 2a that the OCV of the DMFC with thin catalyst is lower (0.177 V) than that with thick catalyst (0.216 V). The OCV can be affected by two factors: Concentration of reactants and electrical insulation [1]. It is then thought that, as expected from the difference of charge transfer resistances, such pore-stuffing effect also blocked the methanol crossover, thereby finally increasing the OCV. Interestingly, in spite of the high starting voltage for thicker electrode case, the final performance is turned around as the current density increases, meaning the charge transfer resistance is a dominant factor as mentioned.

**Figure 3:**
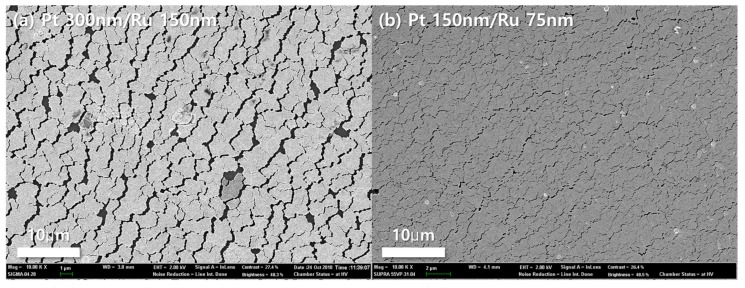
FE-SEM images of the surfaces with different catalysts thickness. (**a**) 150 nm thick Ru on 300 thick Pt, and (**b**) 75 nm thick Ru on 150 nm thick Pt.

The performance difference between thick and thin catalyst layers could also be confirmed by surface morphology in Figure 3. In the case of the thick catalyst layer, as shown in Figure 3a, cracks and delamination of catalysts were seen. Such cracks and delamination came from the expansion coefficient difference between the membrane and catalysts layer. Nafion^®^ 117 membranes must be activated prior to characterizations. However, during activation process, Nafion^®^ membrane absorbed large amounts of water and expanded in volume. At that time, defects occurred in the catalyst layer and the thicker the catalyst layer is, the more severely affected by the volume change [35]. When the catalysts were deposited on the membrane by sputtering, the triple phase boundaries (TPBs) are formed only at the interface between the electrolyte and the electrode. Therefore, due to such cracks and delamination, the thick catalysts layer MEA had less TPBs than the thin one, and as a result, the charge transfer resistance was increased, as shown in Figure 2b.

**Figure 4:**
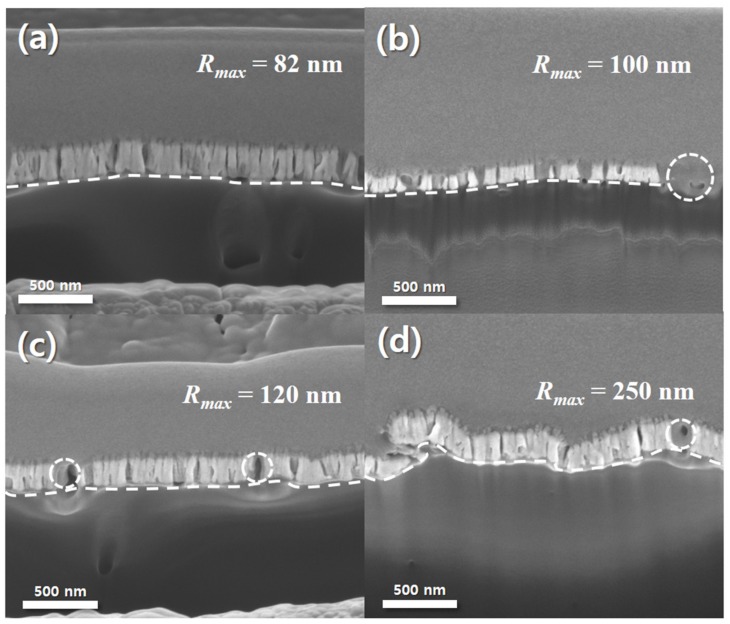
FIB-SEM images of the sputtered Pt and Ru on (**a**) pristine membrane, and roughened membranes by (**b**) 4000, (**c**) 2000, and (**d**) 600 grit sand papers. The measured R_max_ for four surfaces are 82, 100, 120, and 250 nm for (**a**), (**b**), (**c**), and (**d**), respectively.

Although not thoroughly investigated, since 150 nm thick Pt and 75 nm thick Ru was found to give a higher electrochemical performance in Figure 2, such sputtered catalyst was applied similarly to roughened Nafion^®^ 117. Figure 4 presents images of FIB images of the bi-layered Pt–Ru catalysts on anode side of Nafion^®^ 117. As shown in Figure 4, all MEAs have very similar thickness of bi-layered Pt–Ru catalysts (150 nm thick Pt and 75 nm thick Ru). It means that catalyst-coated layers (CCLs) were successfully prepared on Nafion^®^ 117 MEAs by sequential sputtering process without any complicated solution-based spray processes. In addition, it is clearly observable that the roughness of CCLs are successfully varied in Figure 4. The maximum height roughness (R_max_) for pristine, 4000, 2000, and 600 grit roughened CCLs are 82, 100, 120, and 250 nm, respectively. Here, the surface of the MEA which is marked with the white dashed line in each image in Figure 4 becomes rougher as the roughness of sandpapers (4000, 2000, and 600 grit) which were used in pre-treatment becomes rougher from Figure 4a–d. Therefore, it is confirmed that the interface length between Pt catalysts and Nafion^®^ 117 was increased as the roughness of MEA was increased.

**Figure 5:**
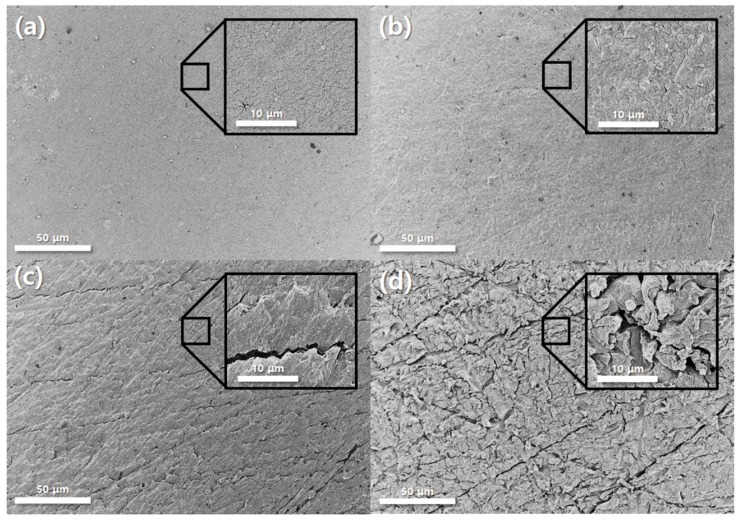
FE-SEM images of the surfaces of sputtered Pt–Ru on (**a**) pristine, (**b**) 4000, (**c**) 2000, and (**d**) 600 grit roughened Nafion^®^ 117 membranes. The insets in each image indicate the magnified surface morphology.

The surface morphologies of the bi-layered Pt–Ru catalysts on Nafion^®^ 117 were investigated further in order to enunciate the relation between the roughness and the electrochemical performance, as shown in Figure 5. All MEAs show rough and porous surfaces in spite of the deposited 250 nm thick sputtered Pt–Ru catalysts. Especially, it seems that the porosity of the surface of a MEA is slightly enlarged as the surface roughness increases. Here, it is noted that the surface morphology of the thin film fabricated by sputtering process is strongly dependent on both the surface roughness of substrate and deposition conditions of sputtering [36,37,38,39]. When considering that the deposition process of bi-layered Pt–Ru catalysts is identical for all MEAs, such differences of the porosities is mainly attributed to the roughness of the MEAs. It is also noticed that the size of pores is <1 μm, meaning that such morphologies would not disturb the diffusion of any reactants (Air and methanol) of the DMFC.

**Figure 6:**
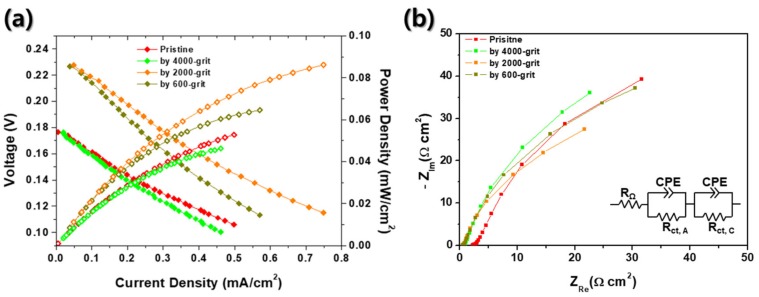
(**a**) Current–voltage and current–power curves and (**b**) EIS spectra of passive DMFCs with four different MEAs: Pristine, roughened by 4000, 2000, and 600 grit.

Finally, the electrochemical characteristics of the passive DMFCs with various roughness MEAs were investigated, as shown in Figure 6. In these evaluations, we used 4 M methanol solution to pursue the simplification of the DMFC systems for portable applications as mentioned above [9]. Here, the grit numbers (4000, 2000, and 600) are the levels of surface roughness of sandpapers which are used in pretreatment of MEAs before the sputter process. According to the Nernst equation, the theoretical OCV of a DMFC is 1.199 V compared to RHE [1]. However, the OCVs indicated in Figure 6 are significantly low regardless of surface roughness of MEAs, which is same as the result of Figure 2a. The OCVs are 0.18, 0.23, 0.23, and 0.18 V for a pristine, 4000, 2000, and 600 grit roughened MEA, respectively. Here again, the low OCVs of DMFCs can be explained by the methanol crossover in electrolyte membranes, as seen in Figure 2a [21]. Methanol crossover from anode to cathode causes the methanol oxidation reactions at the cathode side of the fuel cell. It would end up lowering the OCVs of DMFCs significantly. In this study, in order to prevent the drop of OCV, Nafion^®^ 117 was used as an electrolyte membrane to reduce the methanol crossover through electrolyte because of its sufficient thickness than other electrolyte membrane generally used for PEMFCs (178 μm thick for Nafion^®^ 117 compared to <50 μm thick for normal PEMFCs) [40]. Here, the custom made DMFC of this study shows no discernible defects (no leakage of methanol solution from a chamber). Therefore, we could first conclude that these considerably low OCVs were caused mainly by two reasons: Lack of electrochemical catalyst and insufficient TPBs at the interface between Nafion^®^ 117 and bi-layered Pt–Ru catalysts, which are also the case in Figure 2a. Compared to the general Pt/C- or Pt–Ru/C-sprayed MEAs, 150 nm thick sputtered Pt and 75 nm thick sputtered Ru are extremely lower than the MEAs fabricated by spraying [41]. Theoretical OCV means that the difference of electrical potential at open circuit is measured without any ohmic losses generated from electrical resistances. In real cases, however, enough current is required to measure OCVs of fuel cells to overcome contact resistances between components of evaluation system and the resistances of external wires. In order to generate enough current from the fuel cell, both sufficient anodic and cathode electrochemical reactions should be accompanied with. In other studies, all MEAs have the loading of Pt in normal range (≥1.0 mg_Pt_/cm^2^) [42,43,44,45]. However, in this study, if the Pt layer was 1 cm^2^, 150 nm thick, and super-dense (no pores), Pt loading would be 0.32 mg/cm^2^. In addition, Pt/C catalyst, which was often used as catalyst for DMFCs, was generally applied together with Nafion^®^ solution to make mixed ionic-electronic conductor (MIEC) to increase TPBs. However, catalysts sputtered MEAs only had TPBs at the interface between the electrolyte and the electrode. Thus, it is thought that the significantly low OCVs in Figure 6 is due to insufficient loading of Pt and deficient TPBs.

Interestingly, there are differences between performances of DMFCs with four different MEAs. According to Figure 6a, the DMFC with 2000 grit rubbed MEA has the highest performance among all samples, which is an OCV of 0.23 V and power density of 0.086 mW/cm^2^. Other fuel cells have relatively lower performances, which are 0.053 mW/cm^2^ for a pristine MEA, 0.065 mW/cm^2^ for a 600 grit rubbed MEA, and 0.046 mW/cm^2^ for a 4000 grit rubbed MEA. These results show the strong dependence between surface roughness of MEAs and performance of fuel cells. Accordingly, it can be confirmed that Figure 6a depicts the relations between surface roughness and performances of fuel cells. As shown in Figure 6a, the peak power density and OCV increase as the roughness of an electrolyte membrane increases at low roughness range. However, excessive roughness of MEA causes the reduction of performance and OCVs. It is speculated that the inordinate surface roughness of the electrolyte membrane could bring about the electrical disconnection between sputtered catalyst themselves (Figure 4 and Figure 5). Or the contact area between the sputtered catalyst layer and GDL could be deteriorated due to the highly roughened architecture of the electrolyte membrane. According to the EIS results indicated in Figure 6b, the charge transport resistance is a dominant factor in each case. Although the charge transport resistance does not appear as a semicircle on the Nyquist plot, it can be seen that it is extremely larger than ohmic resistance (<2.2 Ω·cm^2^). Additionally, the size of charge transport resistance can be compared for each MEA, and it can be seen that it has the lowest charge transport resistance at 2000 grit MEA, which has the best performance. It was thought that the roughened MEA had increased the TPBs, thereby decreasing the charge transport resistance.

Further investigations about the relation between the roughness of the electrolyte membrane and the electrochemical performance are required to find out the deep science inside the passive DMFCs and their sputtered catalysts. It could be the optimization of the structures of sputtered layers, thicknesses, and applications of proper GDLs. However, the findings in this study suggest two meaningful results: Sputtered Pt and Ru can be used as an electro-catalyst for passive DMFCs and their structure (thickness of Pt/Ru and roughness of an electrolyte membrane) is a significant parameter affecting the performance of the DMFCs.

## 4. Conclusions

In this study, Pt and Ru were sputtered directly on an electrolyte membrane (Nafion^®^ 117) and it was applied as an MEA for passive DMFCs for the first time. Especially, the influence of the surface roughness of an electrolyte membrane on electrochemical characteristics of passive DMFCs was investigated. In addition, it was operated by using a methanol solution of very high concentration (4 M). As a result, bi-layered Pt–Ru catalysts layer was successfully fabricated by sputtering and surface roughness of an electrolyte membrane was precisely controlled using sandpapers with different roughness level, which were confirmed by SEM images. The performance of the passive DMFCs with the as-prepared MEAs were improved as the roughness of MEA was increased. However, excessive roughness induced the deterioration of the performance. The passive DMFC with the optimal roughness of the electrolyte membrane improved the OCV by 22% and enhanced performance compared to a fuel cell with a pristine MEA by 38%. Although further investigation such as the optimization of the structure and thickness of Pt and Ru, finding proper GDLs, and so forth is required to further improve this type of MEAs and DMFCs, we believe that results of this study can contribute to the reduction of novel metals (Pt and Ru) by using sputtering process and resulting increased possibility of the commercialization of the passive DMFCs.
